# Advanced Biomaterials and Techniques for Oral Tissue Engineering and Regeneration—A Review

**DOI:** 10.3390/ma13225303

**Published:** 2020-11-23

**Authors:** Anamaria Matichescu, Lavinia Cosmina Ardelean, Laura-Cristina Rusu, Dragos Craciun, Emanuel Adrian Bratu, Marius Babucea, Marius Leretter

**Affiliations:** 1 Department of Preventive Dentistry, Community and Oral Health, “Victor Babeș” University of Medicine and Pharmacy Timisoara, 2 Eftimie Murgu Sq., 300041 Timisoara, Romania; matichescu.anamaria@umft.ro; 2 Department of Technology of Materials and Devices in Dental Medicine, “Victor Babeș” University of Medicine and Pharmacy Timisoara, 2 Eftimie Murgu Sq., 300041 Timisoara, Romania; 3 Department of Oral Pathology, “Victor Babeș” University of Medicine and Pharmacy Timisoara, 2 Eftimie Murgu Sq., 300041 Timisoara, Romania; laura.rusu@umft.ro (L.-C.R.); craciun.dragosi@gmail.com (D.C.); babucea.marius@gmail.com (M.B.); 4 Department of Implant Supported Restorations, “Victor Babeș” University of Medicine and Pharmacy Timisoara, 2 Eftimie Murgu Sq., 300041 Timisoara, Romania; 5 Department of Prosthodontics, “Victor Babeș” University of Medicine and Pharmacy Timisoara, 2 Eftimie Murgu Sq., 300041 Timisoara, Romania; mariusleretter@yahoo.com

**Keywords:** regenerative medicine, regenerative dentistry, tissue engineering, stem cells, biomaterials, scaffolds, growth factors, additive manufacturing, 3D printing

## Abstract

The reconstruction or repair of oral and maxillofacial functionalities and aesthetics is a priority for patients affected by tooth loss, congenital defects, trauma deformities, or various dental diseases. Therefore, in dental medicine, tissue reconstruction represents a major interest in oral and maxillofacial surgery, periodontics, orthodontics, endodontics, and even daily clinical practice. The current clinical approaches involve a vast array of techniques ranging from the traditional use of tissue grafts to the most innovative regenerative procedures, such as tissue engineering. In recent decades, a wide range of both artificial and natural biomaterials and scaffolds, genes, stem cells isolated from the mouth area (dental follicle, deciduous teeth, periodontal ligament, dental pulp, salivary glands, and adipose tissue), and various growth factors have been tested in tissue engineering approaches in dentistry, with many being proven successful. However, to fully eliminate the problems of traditional bone and tissue reconstruction in dentistry, continuous research is needed. Based on a recent literature review, this paper creates a picture of current innovative strategies applying dental stem cells for tissue regeneration in different dental fields and maxillofacial surgery, and offers detailed information regarding the available scientific data and practical applications.

## 1. Introduction

The traditional standard techniques based on replacing missing or deteriorated tissue with autologous grafts from living donors or even cadavers are still used in dentistry as well as in other medical fields, despite their disadvantages, such as risk of infections and rejection following the transplantation procedure. An innovative alternative is provided by regenerative medicine, which aims to regenerate, repair, or replace tissues and to ensure restoration of their impaired function by combining tissue engineering with the self-healing ability of humans. In vitro engineering of tissues and organs involves the emerging field of biotechnology in a multidisciplinary approach together with medicine, materials science, cell and molecular biology, bioengineering, and genetics [1].

Tissue engineering is a term associated with regenerative medicine and is distinct in its focus on aspects regarding the engineering and manufacturing of replacement tissue, but regenerative medicine and tissue engineering are often treated as a single field of interest in the literature. Tissue engineering aims to create functional tissue or even organs using patients’ own cells, offering an alternative method to grafts or transplants. This approach is being increasingly used in dental and maxillofacial reconstruction medicine, providing a new option for the reconstruction of teeth, periodontium, bones, oral mucosa, conjunctiva, skin, temporomandibular joint, both bone and cartilage as well as nerves, muscles, tendons, and blood vessels of the oral and maxillofacial area [2].

**Figure 1:**
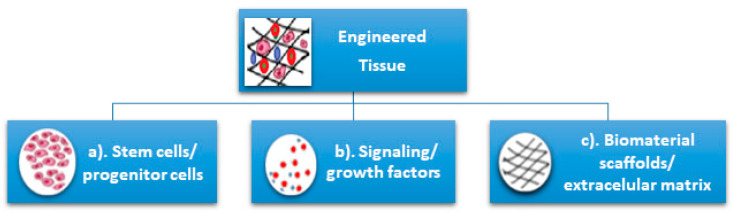
Classical pillars of tissue engineering: (**a**) the cells (stem cells/progenitor cells), (**b**) the signaling/growth factors, (**c**) the biomaterial scaffolds/extracellular matrix.

Tissue engineering can be used to regenerate tissue for specific defects, which represents a major advantage compared with other current treatments which have numerous disadvantages for patients like loss of sensorial and motor functionalities of craniofacial structures due to prosthetic alloplastic materials, high risk of infection, inflammation, requirement for lifelong immunosuppression, or unpredictable compatibility with the donor in the case of autologous grafts. Additionally, the unlimited available bioengineered resources do not require immunosuppression [3]. Tissue engineering is classically based on three pillars: (a) the cells (stem cells/progenitor cells), responsible for synthesizing the new tissue matrix; (b) the signaling/growth factors necessary to promote and facilitate the functionalities; (c) the biomaterial scaffolds, necessary for cell differentiation, multiplication, and biosynthesis, that act as an extracellular matrix (ECM) (Figure 1).

Cells communicate with their environment using different components to regenerate tissues by combining human cells with specific scaffold biomaterials. The biomaterial scaffolds provide templates for tissue regeneration and guide new tissues in their growth [4,5]. A successful approach in tissue engineering and regeneration implies that the combination of these three principles must be able to replace the damaged tissue and enable its function similarly to the original tissue or must be able to stimulate regeneration of the original tissue [6,7]. Several kinds of cells have been used in tissue engineering and regenerative medicine as reported in clinical studies, including stem cells, fibroblasts, chondrocytes, and keratinocytes originating from the same patient, another human, or animals [8].

The aim of this narrative review article is to approach this broad-spectrum subject in view of the literature from recent years specifically on the topic of potential orofacial stem cell usage in regenerative dentistry, both for hard and soft tissues. A large literature survey was performed on this topic in free-access digital archives of full-text articles (PubMed, Medline, Web of Science, and Google Scholar), with articles published between 2010–2020 being considered. More than 300 articles were referenced, with over 50% published in the last five years. The keywords used for searching were “regenerative dentistry”, “tissue engineering”, and “orofacial stem cells”. A specific search was performed to identify clinical studies involving the application of dental stem cells for tissue regeneration in endodontics, periodontics, and maxillofacial surgery.

## 2. Stem Cells, Biomaterials, and Scaffolds for Oral Tissue Engineering and Regeneration—Types, Sources, and Technologies

### 2.1. Orofacial Stem Cells

Stem cells (SCs) are defined as primitive, unspecialized, and pluripotent cells of the human body characterized by two major properties: production of other new stem cells and multidirectional differentiation into cells with a specific functionality, such as bone cells, skin cells, and blood cells [8,9]. Their presence was first reported in bone marrow [10].

SCs have powerful potential in medicine; their study has revealed important information about the complex processes of human body development. Due to these abilities, SCs have attracted interest regarding their use in the regeneration, repair, and functionality improvement of degenerated or injured tissue using implants of engineered tissue as well as biohybrid organs. The strategies involving the use of stem cells for tissue regeneration can be optimized using bioactive scaffolds or by adding various growth factors [11].

Considering their origin, physiological stem cells include embryonic stem cells (ESCs) from embryos and adult stem cells (ASCs) from adult tissue. Other types of stem cells are the perinatal stem cells, from amniotic fluid, and induced pluripotent stem cells (iPSCs) [12], obtained by transforming regular ASCs under genetic reprogramming. iPSCs, which are generated directly from a somatic cell, were pioneered by Yamanaka, in 2006. Shinya Yamanaka’s discovery was awarded with the 2012 Nobel Prize, jointly with Sir John Gurdon, who, in 1962, demonstrated that the specialization of cells is reversible. The immature cell nucleus in an egg cell of a frog was replaced with the nucleus from a mature intestinal cell. This modified egg cell developed into a normal tadpole, proving that the DNA of the mature cell still had all the information needed to develop all cells in the frog [13]. More than 40 years later, Shinya Yamanaka discovered how intact mature cells in mice could be reprogrammed to become PSCs, able to develop into all types of cells in the body, by introducing only a few genes [14,15,16].

The ESCs are present in the blastocyst and can be differentiated into all types of cells, and are therefore pluripotent. Various postnatal tissues present ASCs for their normal renewal as well as regeneration or injury healing. Recent research in tissue engineering and regenerative medicine has demonstrated that SCs can be widely used in dentistry, more so than synthetic materials because teeth are a rich source of SCs [17]. Mesenchymal stem cells (MSCs) are a type of ASC of great importance in regenerative medicine due to their responsibilities in tissue repair and growth, cell substitution, and wound healing due to physiological or pathological causes. MSCs can be isolated especially from bone marrow and adipose tissue, but also from other various human tissues like the placenta, amniotic fluid, liver, umbilical cord, synovial membrane, skin, muscle, and dental tissues [18].

**Figure 2:**
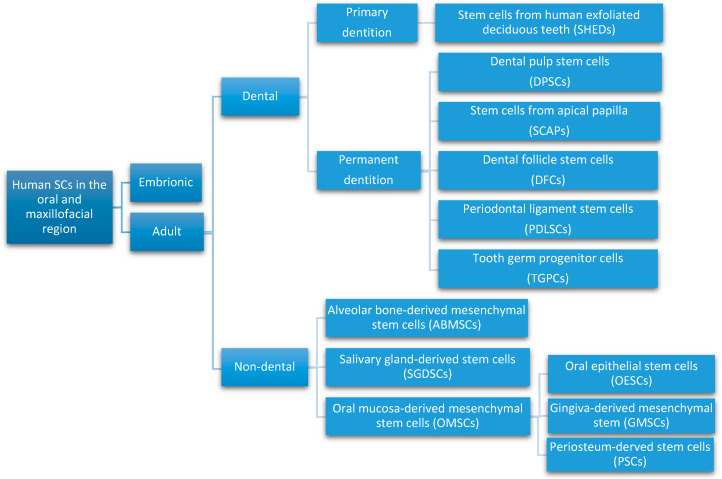
Types of human SCs in the oral and maxillofacial region.

Different types of SCs obtained from oral and maxillofacial tissues, with similar in vitro properties as bone marrow-derived MSCs, are being defined as multipotent stromal cells. They are able to differentiate into different types of cells like chondrocytes, myocytes, osteoblasts, and adipocytes. Recently, the immunomodulatory properties of MSCs have been reported, which enable their clinical use in the treatment of inflammatory conditions [19]. Considering their location in the oral and maxillofacial region, the ASCs are grouped in two major categories: dental and non-dental [20] (Figure 2).

The easy access, proliferation capacity, and multidirectional in vivo/in vitro differentiation makes orofacial SCs an important source of SCs for use in regenerative dentistry and medicine. Therefore, their potential clinical application in dentistry or other medical fields is diverse.

- Dental pulp stem cells (DPSCs), the first human dental MSCs found inside teeth, are considered a significant source for future regenerative procedures both in dental and general medical applications [21]. DPSCs are isolated from the dental pulp of primary or permanent teeth. Their high capacity for in vitro differentiation includes odontoblast, osteoblast, myoblast, adipocyte, dentin–pulp, cardiomyocyte, neuron-like cell, and hepatocyte-like cells, whereas in vivo, they are limited to only adipocytes, endotheliocytes, and myofibers [8,22,23].
- Periodontal ligament stem cells (PDLSCs), present on alveolar bone surfaces and the root, play a specific role in cementum or periodontal ligament (PDL) tissue regeneration. They are capable of giving rise to mesenchymal cell lineages to produce in vitro osteoblast-like cells, cementum tissue, Sharpey’s fibers, adipocytes, and collagen-forming cells [17,24].
- Stem cells from apical papilla (SCAPs) are mesenchymal formations. They can be found within immature roots and isolated from the immature permanent apical papilla. SCAPs are good sources of and cause apexogenesis. They have a higher capacity to proliferate than DPSCs, being the first option for tissue regeneration. SCAPs represent a promising source of SCs, as they can differentiate into various lineages of cells, such as odontogenic, chondrogenic, osteogenic, adipogenic, neurogenic, and hepatogenic cells [25].
- Dental follicle stem cells (DFCs) are sourced from the dental follicle, which is loose connective tissue surrounding the developing tooth germ [17]. DFCs can differentiate osteoblast, cementoblast, alveolar bone, dentin-like tissues, PDL, cementum, adipocyte, chondrocyte, cardiomyocyte, and neuron-like cell. Their regenerative potential is highlighted by clinical applications in periodontal and neural tissue regeneration, tooth root regeneration, and bone defects [17,20,26,27].
- Tooth germ progenitor cells (TGPCs) are obtained from the dental mesenchyme of the human third molar germ in the late bell stage of tooth development. Studies on TGPCs have demonstrated their high proliferation activity and capacity to differentiation into adipogenic, chondrogenic, osteogenic, odontogenic, and neurogenic tissue [28,29]. In addition, TGPCs can differentiate into hepatocytes in vitro [25,30] and are able to form tube-like structures, possibly evidence of vascularization [31].
- Stem cells of human exfoliated deciduous teeth (SHEDs), obtained from exfoliated deciduous teeth, have higher proliferation capacity than DPSCs and the capability to differentiate into many more different body tissues than other types of SCs, including into adipocytes, osteoblasts, odontoblasts, neural cells, hepatocytes, and endothelial cells. SHEDs have a high proliferation capacity, high multipotency, immunosuppressive ability, and minimal risk of oncogenesis [32]. The major disadvantage of SHEDs is that an incomplete pulp-dentin-like complex is formed in vivo [17].
- Alveolar bone-derived mesenchymal stem cells (ABMSCs), isolated from the human alveolar bone, are a more convenient tissue source of MSCs and have the ability of multipotent differentiation into osteoblasts, adipocytes, and chondroblasts. In addition, they can induce ectopic bone formation in vivo [19].
- Salivary gland-derived stem cells (SGDSCs) are isolated from human salivary glands. The regeneration of salivary gland function with SGDSCs is still being investigated, though certain studies have already concluded that progenitor cells isolated from stromal tissue can be guided to differentiate into osteoblasts, chondrocytes, and adipocytes [33].
- Oral mucosa-derived mesenchymal stem cells (OMSCs), include oral epithelial stem cells (OESCs), gingiva-derived mesenchymal stem cells (GMSCs), and periosteum-derived stem cells (PSCs). SCs within the mucosa lining the oral cavity can be isolated from normal or inflamed gingiva, from attached and free gingiva, and from hyperplastic gingiva. OMSCs can differentiate into different mesenchymal lineages and have immunomodulatory properties [33].

### 2.2. Biomaterials and Scaffolds for Oral Tissue Engineering

In dental tissue regeneration, scaffolds and biomaterials are essential elements. They are used as attachment sites for regenerative cells from the surrounding tissues, as a template for tissue regeneration, as a source of implantable odontogenic cells with the capability to differentiate required cell type, and as bioactive molecules, especially growth factors that intensify the regenerative capability [34,35].

Biomaterials, natural or synthetic, alive or lifeless, are being defined as materials that interact with biological systems. They are often used in medical applications to augment or replace a natural function. Based on their biocompatibility, biomaterials are classified as bioactive, biotolerant, biodegradable, and bioinert [36].

Bioactive materials, by stimulating the biological response, may lead to osteogenesis by making strong chemical bonds. They are being classified into osteoconductive (hydroxyapatite and β-tricalcium phosphate), which stimulate bone growth along the surface, and osteoproductive (bioactive glasses), which are capable of stimulating the growth of new bone away from the bone/implant interface [36].

Biotolerant materials (polymers and most metals) are being well accepted by the host, but separated from the host tissue by the formation of a fibrous tissue, which is induced by the release of ions, corrosion products, and chemical compounds from the implant.

Biodegradable materials (polymers, such as polyglycolic and polylactic acids, and their co-polymers [37], ceramics as calcium phosphates [38], and magnesium) as biodegradable metal dissolve in contact with body fluids, the dissolution products being eliminated via the kidneys, without noticeable effects to the host. Biodegradable materials are used commonly used for surgical sutures, tissues in growth materials, and controlled drug release [36].

Bioinert materials (titanium and its alloys) are stable in the human body, and do not react with body fluids or tissues. Generally, bioinert materials are encapsulated by fibrous tissues, similar to biotolerant materials; however, in certain situations, they can develop structural and functional connection with the adjacent bone [39].

The most common approach in tissue engineering involves seeding cells onto a biomaterial matrix using a scaffold.

A wide variety of biomaterials, such as natural organic, synthetic organic, or even inorganic materials, is used for regeneration in oral and maxillofacial area, each of them having advantages and disadvantages. The natural organic materials include peptides (collagen or gelatin) and polysaccharides (alginate, chitosan, agarose). Frequently used synthetic organic materials include poly(lactic acid) (PLA), poly(caprolactone) (PCL), poly(lactic-co-glycolic acid) (PLGA), and poly(glycolic acid) (PGA) [36].

The most commonly used inorganic materials are bioactive ceramics which include glasses or calcium phosphates (hydroxyapatite, β-tricalciumphosphate), which have been extensively studied as bone replacement materials, and cementitious systems of calcium phosphate or calcium silicate [40].

Bioactive ceramics are strongly chemically bonded with bone tissues via chemical reactions [40]. Hydroxyapatite (HA), bioactive and non-degradable, is characterized by chemical and structural similarity to bone minerals. β-tricalcium phosphate also has a chemical composition similar to bone, and has higher in vivo rates of biodegradation compared to hydroxyapatite. The degradable bioactive ceramics are characterized by gradually degradation, in order to assist as scaffolds or replace the host tissue [40].

Polymers have been widely studied for medical applications, including bone tissue engineering [41]. From a biomedical perspective, polymers and co-polymers can be divided into two classes, biodegradable and biotolerant.

Biodegradable polymers, synthetic and natural, are suitable for additive manufacturing of scaffolds for tissue engineering [42]. The degradation of polymers, enzymatical or hydrolytical, is of most importance for this application. Natural polymers (chitosan, alginate, collagen, gelatin), frequently used as bioinks, are subject to enzymatic degradation, due to the microorganisms present in the biological environment [43].

The rate of enzymatic degradation varies upon the availability and concentration of respective enzymes. Hydrolytical degradation is related to synthetic polymers, and involves cleavage of hydrolytically sensitive bonds in the polymer, with consequent bulk or surface erosion, important in determining the best choice for a certain application [44].

Surface erosion offers several benefits for bone tissue engineering, such as retention of mechanical integrity, enhanced bone ingrowth, and ensures that the scaffold is gradually replaced by bone tissue [45].

PGA, PLA, PLGA, and PCL are hydrolytically degradable polymers [46].

PGA is usually used for short-term tissue engineering scaffolds and as fillers, because its rapid degradation and insolubility [47].

PLA, when mixed with glycolic acid, forms the copolymer PLGA, which is one of the most investigated degradable polymer for biomedical applications. Its great cell adhesion and proliferation properties recommend it as an excellent choice for tissue engineering [48,49].

Polymers can be processed to offer porous structures capable of facilitating the transportation of growth factors (nutrients as well as anabolites and catabolites) and are of interest due to their controllable degradation [41].

Recently, composite materials are being increasingly used due to their properties that result from the combination of both organic and inorganic elements. The most recent studies on this subject have considered the targeted and scaffold-assisted regeneration of enamel, dentin, and cementum [35].

An essential factor in tissue engineering is the scaffold. It offers a surface upon which cells adhere, multiply, thrive, and produce the ECM of proteins and saccharides that create the living tissue. Cells are expanded in culture and then transferred to the scaffold. The composition of the scaffold material and its internal architecture (dimensions of the struts, walls, pores, or channels) modulate and control the biological properties of the cells [50].

Generally, the scaffold materials must be biocompatible, biodegradable, porous, and without toxic metabolites. In particular, in dental regeneration, biomaterials must be suitable for the specific environment characteristics of the oral cavity considering pH, temperature, the presence of microorganisms, and the effect of mastication forces. To achieve these properties, most designed scaffolds deliberately mimic the structure of the natural ECM [36].

The number of suitable materials for fabricating scaffolds is limited by their biocompatibility, as they must accommodate the encapsulated cells and the recipient’s body. Because of poor biocompatibility, scaffolds can generate aggressive in vivo foreign-body reactions, necessitating the development of smart immunomodulatory biomaterials that ensure the tolerance of foreign scaffolds by the host or regulating the immunological microenvironments to ensure cell survival [49].

The behavior of cells after adhesion to the scaffold is affected by pore shape, volume, size, and geometry. Different pore sizes can affect the extracellular matrix. Porosity and interconnectivity are important for the ingrowth of surrounding tissues [51]. Open and interconnected pores allow oxygen and nutrients to be transported into the interior and eliminate the waste generated by cellular metabolism [52].

A wide range of advanced smart biomaterials and constructs with intelligent properties and functions have recently been developed to improve tissue repair and regeneration processes [5]. Smart scaffolds incorporate bioactive molecules and nanoparticles and their physical and chemical properties are tailored as needed [53,54]. Their role is to improve the interactions with cells by enhancing the osteogenic differentiation for bone repair and to generate a better response to the surrounding environment [55] and include [5]:

- Smart scaffold constructs with stem cells for bone tissue engineering
  - Biomimetic and bionic smart scaffolds, such as biomimetic porous PLGA microspheres coupled with peptides prepared to mimic the composition and structure of natural tissues [56].
  - Immune-sensitive smart scaffolds, such as an amino-functionalized bioactive glass scaffold developed to investigate its effects on MSCs, bone marrow, and macrophages [57]. β-tricalcium phosphate has been used to coat Mg scaffolds, and modulate its detrimental osteoimmunomodulatory properties [58].
  - Shape-memory smart scaffolds, such as bone morphogenetic protein2-loaded shape-memory porous nanocomposite scaffold, consisting of chemically crosslinked poly(ε-caprolactone) and hydroxyapatite nanoparticles, used for the repair of bone defects, displayed shape-memory recovery [59].
  - Electromechanical-stimulus smart scaffolds. Piezoelectric poly(vinylidene fluoride-trifluoroethylene) (PVDF-TrFE) was fabricated into flexible, 3D fibrous scaffolds. These have the ability to stimulate MSCs differentiation and tissue formation [60]. An electrospun PVDF-TrFE fiber scaffold containing zinc oxide nanoparticles was able to promote the adhesion and proliferation of human MSCs and also enhance the blood vessel formation [61].
- Smart drug delivery for bone tissue engineering
  - Stimuli-responsiveness tunable drug delivery systems. These materials can change their properties as response to an endogenous and/or exogenous stimulus; thus, delivering the required amount of drug on-demand [62]. Polymers and hydrogels are used [63,64]. A highly porous, pH-responsive bacterial cellulose-g-poly(acrylic acidco-acrylamide) hydrogel was developed as an oral controlled-release drug delivery carrier [64]. A poly(ethylene glycol) hydrogel, loaded with drugs by β-eliminative linkers, demonstrated tunable capability in drug release [65]. Farnesol-loaded nanoparticles, composed of 2-(dimethylamino)ethyl methacrylate, butyl methacrylate, and 2-propylacrylic acid are characterized by a pH-responsive drug release capability [66].
  - Smart multifunctional nanoparticle-based drug delivery systems: mesoporous silica nanoparticles, bone-forming peptide-1-laden MSNs encapsulated into arginine-glycine-aspartic acid-treated alginate hydrogel [67].
  - Biomimetic drug delivery systems: hydrogels, liposomes, micelles, dendrimers, polymeric carriers, and nanostructures [68,69].
- Smart biomaterials and constructs to promote dental and periodontal regeneration, such as bilayered PLGA/calcium phosphate constructs [70] and tri-layered nanocomposite hydrogel scaffold: alveolar bone phase of chitin-PLGA/nanobioactive glass ceramic (nBGC)/platelet-rich plasma derived growth factors, PDL phase of chitin-PLGA/fibroblast growth factor, and cementum phase of chitin-PLGA/nBGC/cementum protein 1 [71].
- Smart dental resins that respond to pH to protect tooth structures, such as dental composites containing nanoparticles of amorphous calcium phosphate and tetracalcium phosphate [72].
- Smart pH-sensitive materials selectively inhibit acid-producing bacteria, and include cationic poly(phenylene vinylene) derivative, pH-sensitive quaternary pyridinium salts, for which the antibacterial potency can be controlled by varying the pH [73,74].
- Smart resins that modulate the oral biofilm composition: quaternary ammonium methacrylates such as 12-methacryloyloxy dodecyl pyridinium bromide, methacryloxylethyl cetyl dimethyl ammonium chloride, quaternary ammonium polyethylenimine, and dimethylaminododecyl methacrylate [75,76].
- Smart tailoring of alkyl chain length in quaternary ammonium methacrylates to avoid drug resistance [5,77].

SCs are capable to differentiate into various cell phenotypes based on their lineage and exposure to different environmental stimuli, such as ECM, growth factors, hypoxia, etc. [78]. The growth factor, usually a secreted protein or a steroid hormone, stimulates wound healing, cell proliferation, and occasionally cellular differentiation, and regulates various cellular processes. Cytokines and hormones bind to specific receptors on the surface of the target cells. Growth factors typically act as signaling molecules between cells, thus promoting cell differentiation and maturation [79,80].

The authors experience related to the subject includes tetracycline loaded collagen-carboxymethylcellulose/hydroxyapatite ternary composite materials [81], antiseptic composite materials containing silver nanoparticles, based on collagen, hydroxyapatite, and collagen/hydroxyapatite [82], collagen matrices with lidocaine [83], bone regeneration using synthetic HA, with high porosity and surface area for osteointegration [84,85,86,87].

### 2.3. Additive Manufacturing Technologies for Oral Tissue Engineering

Continuous development of manufacturing technologies enable printing of biofunctional scaffolds similar to the ECM, acting as a microenvironment for cell adhesion, proliferation, and differentiation [88,89].

The additive manufacturing (3D printing) of biomaterials offers promising future perspectives for the field of biomedical engineering [90], especially in regard to patient-specific clinical applications.

Additive manufacturing techniques for medical and tissue engineering purposes can be classified as: techniques which involve printing of live cells along with other materials (3D bioprinting) [91], and non-cellular fabrication techniques.

3D bioprinting, based on the layer-by-layer precise positioning of biological constituents, biochemicals and living cells, facilitates on-demand “printing” of cells, tissues and organs [92,93] for regenerative medicine purposes [94]. Utilizing diverse bioprinting techniques, tissue-engineered constructs can be tailored to obtain desired structures and properties [95,96].

Inkjet bioprinting functions by depositing small ink droplets into a predetermined location. It can be driven by thermal or piezoelectric actuation [97]. In thermal technology, heat-generated, the inflated bubble forces the ink out of the narrow nozzle and onto the substrates. In piezoelectric technology, drops are generated in absence of heat, by the transient pressure from the piezoelectric actuator. The droplets remain directional with regular and equal size [98], but, if used too frequently, this technology can cause damage to the cell membrane and cell lysis.

Laser-based bioprinting consists of a pulsed laser source, a ribbon, and a receiving substrate. The biological material, in liquid form, is irradiated by the laser, evaporates, and reaches the receiving substrate as droplets. Laser-based bioprinting enables high-resolution printing of biological material such as cells, DNA, and peptides [99]. Its drawback is that the use of the pulsed laser source may result in compromised cell viability [100].

Stereolithography bioprinting uses a photo-crosslinking light source to obtain desired patterns. It is highly tunable and prints in a layer-by-layer manner, the bioink from the reservoir being transferred to a movable platform [101].

Pressure-assisted bioprinting uses biomaterials in form of solutions, pastes or dispersions. The material, in form of a filament, is extruded by pressure through a microneedle or a microscale nozzle orifice [102].

Bioink printability has an important role in the fabrication process [103,104]. Besides being biocompatible and biodegradable, bioinks should be deformable and flowable [102]. After printing, the bioink should be stable in order to maintain shape and architecture of the design model [105].

The components of the bioink are polymers, ceramics, hydrogels, and composites, currently used in tissue engineering [106]. Hydrogel inks are much more attractive as bioprinting materials, compared to polymers and ceramics have received much more attention, and novel ink formulations have been designed [107]. Complex, functional, and biocompatible hydrogels can be fabricated using bioprinting technology. Adding different amounts of HA was attempted to a tunable alginate-gelatin hydrogel composite [108], human MSCs being subsequently mixed. Adding HA to the hydrogel resulted in enhanced mechanical properties, recommending it hard tissue reconstruction. No reduction in cell viability was detected [109]. The freeform reversible embedding of suspended hydrogels, a 3D bioprinting technique which deposits and crosslinks different kind of hydrogel inks, has been proven successfully [110].

An important concern when printing SCs—including ESCs, MSCs, and ASCs—is that their activity, including proliferation and pluripotency, may change during the process [111,112]. MSCs were successfully laser-printed for the construction of scaffold-free autologous grafts. The seed cells survived and maintained their ability to proliferate and continue differentiating into the osteogenic lineage [113].

Non-cellular additive manufacturing techniques include (Table 1):

The powder bed fusion methods which use either electron beam or laser to selectively consolidate material powder. The techniques involve spreading material powder over the previous layers, melting and fusing it [114].

The binder jetting technique is similar to the powder bed fusion technique and utilizes material powder that is spread over previous layers. Unlike powder bed fusion, this technique uses a binder as an adhesive for its consolidation [115,116].

The fused deposition modeling technique is based on the extrusion of heated polymer wires through a nozzle tip. The polymer rods are deposited and arranged in a layer by layer fashion [117].

The material jetting technique uses a liquid photopolymer resin that is light-cured. Similar to the material extrusion technique, the material is deposited from a nozzle and cured, defining a cross section. Individual cross sections are consolidated in a layer by layer fashion as the building platform moves in the vertical direction [118].

The vat polymerization technique uses a vat of liquid photopolymer resin, deposited in a layer by layer fashion. The build platform moves (depending on the position of the light source) to create additional layers on top of the previous [119].

These techniques all have their pros and cons and can process different types of biomaterials [120] (Table 1).

**Table 1:** Additive manufacturing methods of biomaterials for oral tissue engineering.

Biomaterial	Type	Fabrication Method	Application	Reference
Hydroxyapatite	Bioactive/non-degradable ceramic	Vat polymerization; powder bed fusion; fused deposition; binder jetting	Bone tissue engineering	[121,122,123,124,125]
Bio glass	Bioactive ceramic	Vat polymerization	Bone tissue engineering	[126]
Calcium silicate	Bioactive ceramic	Powder bed fusion	Tissue engineering	[127]
β-tricalcium phosphate	Bioactive/ biodegradable ceramic	Binder jetting; vat polymerization; fused deposition	Bone tissue engineering	[128,129,130,131,132]
Polycaprolactone	Biodegradable polymer	Powder bed fusion; fused deposition	Bone tissue engineering; cartilage tissue engineering	[133,134,135,136]
Poly(lactic acid)	Biodegradable polymer	Fused deposition	Bone regeneration	[137]
Poly(lactic acid-co-glycolic acid)	Biodegradable polymer	Material jetting; fused deposition	Tissue engineering	[138,139,140,141]

## 3. Regenerative Therapies in Dentistry—Potential Clinical Applications of Dental Stem Cells

Four main groups of defects in the oral area represent the main targets of soft or hard tissue regeneration: maxillofacial defects, periodontal diseases (gingiva inflammation, PDL, alveolar bone, and cementum loss), dental pulpal diseases, and hard tissue defects of the tooth [142]. In addition, tissue engineering and regeneration are oriented toward several applications of dental SCs with the aim of accelerating the healing of oral injury without scar formation [143]. Table 2 lists the potential clinical applications of dental SCs in regenerative dentistry.

**Table 2:** Potential clinical applications of dental SCs in regenerative dentistry.

Type of SCs	Regenerative Dental Applications	References
DPSCs	Mandibular bone defects regeneration, scaffold-based dentin–pulp repair, dentin–pulp tissue regeneration with inflamed pulp, periodontal regeneration, neural tissue regeneration, muscle regeneration, angiogenesis induction, craniofacial skeletal repair	Zhou et al. [11] Zakrzewski et al. [17] Berebichez-Fridman et al. [18] Hollands et al. [22] Tsutsui [23] Sharpe [24] Khazaei et al. [28] Chalisserry et al. [30] Somani et al. [31] Yang et al. [142] Chatzistavrou et al. [144] Bakopoulou et al. [145] Tatullo et al. [146] Potdar et al. [147] Davila et al. [148] Gronthos et al. [149] Beltrão-Braga et al. [150] Verma et al. [151] Almushayt et al. [152] Yoshida et al. [153] Aydin et al. [154] Graziano et al. [155]
PDLSCs	Tooth root regeneration, periodontal tissue regeneration (cementum, PDL), bone regeneration	Zhou et al. [11] Zakrzewski et al. [17] Liu et al. [20] Somani et al. [31] Verma et al. [151] Aydin et al. [154] Kitagaki et al. [156] Hynes et al. [157] Han et al. [158] Maeda et al. [159] Gay et al. [160] Kim et al. [161]
SCAPs	Bone regeneration, tooth root regeneration, dentin–pulp repair, neural regeneration and repair, periodontal regeneration, angiogenesis, tooth regeneration	Zhou et al. [11] Liu et al. [20] Kang et al. [25] Khazaei et al. [28] Somani et al. [31] Bakopoulou et al. [145] Verma et al. [151] Aydin et al. [154] Schneider et al. [162] Nada et al. [163] Miller et al. [164] Wongwatanasanti et al. [165]
DFCs	Bone defects, tooth root regeneration, periodontal tissue regeneration, neural tissue regeneration, enhancement of bone regeneration on titanium implant surfaces in humans	Zhou et al. [11] Zakrzewski et al. [17] Liu et al. [20] Chalisserry et al. [30] Somani et al. [31] Yang et al. [142] Verma et al. [151] Aydin et al. [154] Zhang et al. [166] Shoi et al. [167] Rezai-Rad et al. [168] Honda et al. [169]
TGSCs	Bone repair and cartilage regeneration	Zhou et al. [11] Chalisserry et al. [30] Verma et al. [151] Aydin et al. [154] Caracappa et al. [170] Yalvaç et al. [171] Yalvaç et al. [172] Doğan et al. [173]
SHEDs	Critical-sized craniofacial bone defect regeneration, scaffold-based dentin–pulp regeneration, neural and blood vessel regeneration, tooth root regeneration, tubular dentin	Zhou et al. [11] Liu et al. [20] Sharpe [24] Somani et al. [31] Verma et al. [151] Aydin et al. [154] Jeon et al. [174] Araújo et al. [175] Ma et al. [176] Kunimatsu et al. [177] Ching et al. [178] Miura et al. [179] Martinez Saez et al. [180] Annibali et al. [181] Arora et al. [182]
ABMSCs	Bone defects, periodontal regeneration	Zhou et al. [11] Liu et al. [20] Verma et al. [151] Aydin et al. [154] Caracappa et al. [170] Mason et al. [183] Liu et al. [184] Pekovits et al. [185] Matsubara et al. [186] Park et al. [187] Lim et al. [188] Khazaei et al. [189]
GMSCs	Neural regeneration, periodontal regeneration, cartilage, bone, muscle, oral mucositis, improving the regeneration of craniofacial bone	Liu et al. [20] Chalisserry et al. [30] Grawish [33] Verma et al. [151] Aydin et al. [154] Caracappa et al. [170] Zhang et al. [190] Tomar et al. [191] Tang et al. [192] Wang et al. [193] Marynka-Kalmani et al. [194] Zhang et al. [195]

### 3.1. Regenerative Endodontics

Regenerative endodontic therapy (RET) is defined as “biologically based procedures designed to replace damaged tooth structures, including dentin and root structures, as well as cells of the pulp–dentin complex” [196]. Regenerative endodontics aims to restore normal function of the pulp, by regenerating the dentin–pulp complex damaged by infection, trauma, or developmental anomalies of immature permanent teeth with necrotic pulp. The benefits of regenerative endodontics not only stand in revitalization of the tooth, but also continued root development and, potentially, increasing fracture resistance [197].

Apexification and apexogenesis are clinical procedures closely related to regenerative endodontics [198]. Pulp necrosis in young permanent teeth poses a challenge to clinicians due to the open and underdeveloped apex [199]. The purpose of endodontic treatment, or hermetic sealing of the foramina, can be easily achieved in mature permanent teeth where there is an apical constriction. Because the young permanent teeth do not have an apical constriction, a hermetic seal of the foramina is almost impossible. It traditionally consists of the apexification procedure with calcium hydroxide or a mineral trioxide aggregate (MTA) plug, which stimulates the periapical cells to form a dentin-like substance in the apex region. This process, even if it seals the foramina, does not add to the thickness and strength of the dentin walls, making the root prone to fractures and resulting in a weakened apical barrier [200,201,202]. Apexogenesis, used in case of injured but not necrotic pulp, leaves the apical one-third of the dental pulp in place, to allow complete formation of the root [198].

The first studies on pulp regeneration were conducted by Nygaard-Otsby et al. [203,204]. Intentionally, overinstrumentation was used to induce bleeding from the periapical tissues into the root canal, followed by a short obturation to allow tissue growth into the canal space. The histological examination of the extracted teeth revealed that fibrous connective tissue and cellular cementum formed in the canal space [203]. Later on, Banchs and Trope [205] proposed a revascularization protocol based on the experiments of Kling et al. [206] on implanted teeth, Hoshino et al. [207] on root canal disinfection, and Nygaard-Otsby et al. [204] on blood clots in the canal space.

Regenerative endodontics originates from the revascularization literature, which focuses only on the delivery of blood into the root canal space. It aims to allow its filling with vital tissue as a result of wound healing, but does not include a source of SCs within the apical tissues, their delivery into root canals, and the intentional release and use of local growth factors embedded into the dentin [208].

The American Association of Endodontists’ (AAE) clinical considerations RET define success by three measures [209]: the primary/essential goal, which is the elimination of symptoms and the evidence of bony healing and is the objective of all endodontic treatments; the secondary/desirable goal, which is increased root wall thickness and/or increased root length and, thus, the continuation of root maturation leading to a smaller incidence in root fracture; and the tertiary goal, which is a positive response to vitality testing.

RET represents an extension of root canal therapy, aiming to heal apical periodontitis. Conventional root canal therapy only cleans and fills the pulp chamber with biologically inert material. RET aims to replace live tissue in the pulp chamber and regenerate its normal function, by stimulating its regrowth or by inserting bioactive substances in the pulp chamber [210].

**Figure 3:**
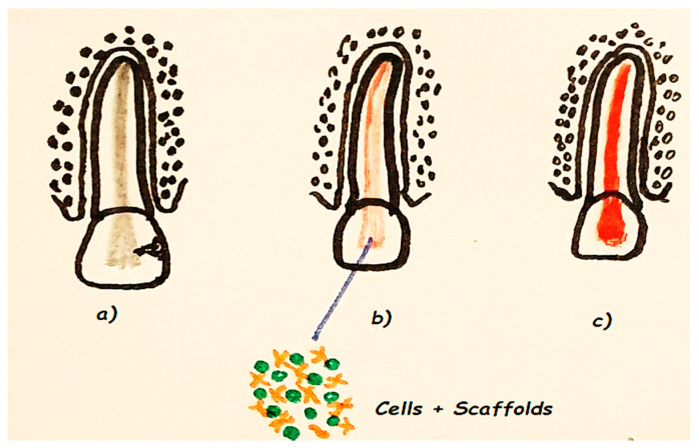
Regeneration of functional pulpal tissue: (**a**) non-vital pulp, (**b**) transplantation of stem cells, (**c**) regenerated pulp.

Previous studies evaluated combinations of SCs, growth factors, and scaffolds that result in histological regeneration of pulp tissues [211] (Figure 3).

ASCs, especially MSCs (DPSCs, SCAPs) are used in RET. Lacerating the apical papilla and subsequently delivering a high local concentration of SCs into the root canal space does not necessarily result in their differentiation into cells of the pulp-dentin complex. Growth factors act as important adjuncts in RET. Histologic signs of tissue repair rather than regeneration may be due to lack of control of endogenous growth factors [212].

SCs are capable of differentiating into odontoblasts, pulp fibroblasts, and other niche cells characteristic of dentin–pulp complex. To ensure the success of RET in the adult, exogenously delivered and/or endogenous growth factors must induce the sprouting of neural fibrils and endothelial cells along with other blood vessel resident cells [213].

Regenerative endodontics is based on adequate disinfection of the root canal system, induction of bleeding through overinstrumentation to create a scaffold for stem cells, and coronal sealing of the blood clot with a biocompatible material, such as MTA [214].

However, certain variables related to patient age, apex diameter, canal instrumentation, disinfection, medication, and coronal seal have to be considered when evaluating RET success.

Even though RET has been used on mature teeth, most of the reported cases are on young patients where pulp necrosis has halted the root maturation process. According to Estefans et al. [215] younger age groups are better candidates for revascularization procedures than older age groups.

In immature permanent teeth, apical diameter is of importance for RET. In cases of a preoperative apical diameter wider than 1 mm, greater root maturation was observed [215]. Nevertheless, apical diameters of 0.5–1.0 mm demonstrated the highest clinical success rate [216]. The pulp tissue regeneration is influenced by the presence of prior infection, which negatively affects the tissue-forming cells as well as SCs in the periapical tissues [217].

The removal of pulp necrotic tissue is vital to the success of pulp regeneration but mechanical removal may be contraindicated because it weakens the already affected dentinal walls [20] and could damage vital tissue remnants in the apical part of the canal [218,219]. According to Lin et al. [220] most of the bacteria are hosted in the apical portion and the biofilm formed on the canal walls penetrates the dentinal tubules. They concluded that, to some degree, mechanical debridement might be necessary to disrupt the biofilm for better chances of root maturation to continue [220], as root-canal-irrigating solutions and intracanal medicaments are not able to completely eliminate bacteria biofilms in infected root canals during root canal therapy [221,222].

Infection prevents regeneration, repair, and SCs activity, so disinfecting the root canal system is crucial to the success of RET [223]. Strategies for optimal disinfection of the pulp space with minimal disruption of the necessary biological factors from dentin, the progenitor cells in periapical vital tissues, and the vascularity, to promote periapical healing as well as soft and hard tissue development after an infectious process are being currently available [214].

After an infection, new tissues cannot form inside the canal space. Only if osteoblasts, cementoblasts, periodontal ligament cells, and endothelial cells can migrate inside the canal is there a chance of developing new tissues.

The chemicals used to disinfect the root canal system have bacteriostatic or bactericidal properties and should not damage healthy tissues, thus lowering the chances of RET success [214,224].

NaOCl is a potent antimicrobial agent that effectively dissolves necrotic and organic tissue [225], which is very effective against biofilm [226,227]. Based on the cytotoxic effect of NaOCl on in vitro survival of SCAPs, a concentration of 1.5% NaOCl is recommended [58,228]. Other studies [229] reported that the SCAPs survival rate is 74% after being exposed to 6% NaOCl followed by 17% ethylenediaminetetraacetic acid (EDTA) and 6% NaOCl once more. The AAE suggests irrigation with NaOCl for 5 min and then with saline or EDTA for 5 min, using a system that lowers the possibility of irrigant extrusion into the periapical space, at about 1 mm shorter than the working length, to maximize the survival rate of SCAPs [230]. Hence, the NaOCl concentrations could be adjusted with some precautions and the SCAP survival rate is not significantly affected [205,218,219,231].

EDTA is a chelating agent used to remove smear layer in conventional root canal therapy [232] and to cause the release of growth factors from dentin matrix in RET [233], resulting in dentin demineralization and its exposure to the released growth factors [230,234]. The use of 17% EDTA resulted in an increased SCAP survival rate as well as partial reversal of the deleterious effects of NaOCl [229]. EDTA conditioning of dentin promoted the adhesion, migration, and differentiation of DPSCs toward or onto dentin [230]. Therefore, a final rinse with EDTA before creation of a blood clot is advised. Release of growth factors from dentin matrix after EDTA treatment was reported in non-infected root canals [233,234]. A residual biofilm may significantly diminish the bioavailability or bioactivity of dentin-matrix-associated growth factors [235]. Dentin-matrix-derived growth factors released after EDTA treatment may signal SCAPs to differentiate into odontoblast-like cells [236].

The use of chlorhexidine (CHX) as canal disinfectant is based on its antimicrobial activity that extends by interacting with the dentin. CHX cannot dissolve tissues and it is not advisable to use it as the only irrigation solution [225,237]. Haapasalo et al. [225] suggested that the initial NaOCl irrigation should be followed by sterile saline and 2% CHX, the role of saline solution being to stop any interactions between NaOCl and CHX.

Intracanal medication between endodontic treatment sessions assists with the control of microbial infection by using different substances as calcium-hydroxide-based and polyantibiotic pastes. It aims to stop microbial proliferation in the root canal system and combine antibacterial and anti-inflammatory properties with the capacity to induce mineralized tissue formation, having beneficial effects on repairing the apical tissues [238].

Traumatized immature permanent teeth with infected necrotic pulp have similar microbial ecology as mature permanent teeth [239], including biofilms formation on the radicular canal walls and bacteria penetration into the canal dentinal tubules [240].

Antibiotics have been used as intracanal medication in root canal treatment since the 1950s [241], but local application of antibiotics in endodontics has been restricted because of the risks of adverse effects. The interest in using a combination of antibiotics has reemerged with the introduction of the triple antibiotic paste (TAP) [238]. The ciprofloxacin, metronidazole, and minocycline TAP [207] is sufficiently potent to eradicate bacteria from the root canal. A double antibiotic paste of metronidazole and ciprofloxacin [218] has also proven its efficacy. Studies have shown that TAP is biocompatible [239] but, unfortunately, antimicrobial combinations can prove to be cytotoxic and increase the risk of adverse effects, and bacterial resistance [239,240]. Augmentin has been shown to kill 100% of the microorganisms isolated from the infected root canal associated with in vitro apical abscess [241]. It acts by inhibiting bacterial cell wall synthesis, only affecting bacterial cells and not human cells, as the latter do not have a cell wall.

Calcium hydroxide is considered the first choice for intracanal medication in RET. It offers good antimicrobial properties, anti-inflammatory activity, consequent stimulation of apical repair, and participation in mineralized tissue formation, inducing differentiation of periodontal ligament cementoblasts and cementogenesis by increasing extracellular calcium levels and tissue compatibility [242]. According to prior studies, dentin is capable of inactivating root canal medication [243,244], thereby limiting the efficacy of calcium hydroxide as an intracanal dressing [245]. Because of its high pH, it can damage the cells that have regenerative capacity [246]. When treated with calcium hydroxide rather than TAP, human apical cells attach to the root dentin walls at a higher rate [247].

Various other materials have been used to induce apexification, such as tricalcium phosphate [248], collagen calcium phosphate [248], osteogenic protein-1 [246], and MTA [246] without affecting root elongation or maturation [246]. The apical plug of MTA and gutta-percha filling has several advantages over calcium hydroxide-induced apexification. MTA is biocompatible, has osteoinductive properties, sets in the presence of moisture, and the treatment can be completed in a single appointment, though it does not strengthen the remaining tooth structure [249].

After disinfection of the canal and resolution of symptoms, RET usually involves lacerating of the periapical tissues to initiate bleeding or the use of platelet-rich plasma (PRP) [250], platelet-rich fibrin (PRF) unmineralized tissue matrices, and synthetic materials like polyglycol or collagen [251,252].

Studies have shown that inducing bleeding into the disinfected canal is an important step in regenerative procedures; a stable blood clot (BC) not only serves as a scaffold but triggers significant accumulation of undifferentiated STCs into the canal space [253] and stimulates cell growth and the differentiation of STCs into odontoblast-like cells [228,253,254,255].

A common problem is the failure to induce apical bleeding or to achieve adequate blood volume in the canal [202,256,257]. In pluri-rooted teeth, this can be achieved by transferring some blood from other roots, but this approach cannot be used for single-rooted teeth. Because of this, researchers have searched for other scaffold options. PRP, PRF, and platelet pellet (PP) are options that have shown promising clinical and radiological results [256]. Cehreli et al. [257] reported the clinical outcomes of PRP, PRF, and PP used in the presence or absence of a BC. PRP, PRF, and PP, even if more expensive than the BC method, can offer a longer exposure to growth factors, and are possibly better scaffolds since they also eliminate the progressing obliteration of the root, a problem found with the BC method [257].

After the scaffold has set and stability has been confirmed, a coronal seal should be placed over the blood clot to serve as an internal matrix. The AAE recommends an MTA layer of approximately 3 mm, followed by a 3–4 mm layer of glass ionomer and a layer of reinforced composite resin [209]. The MTA, which hardens in wet conditions, acts like an antibacterial barrier, but is also associated with teeth discoloration. An alternative to MTA, such as bioceramics and tricalcium silicate cements, should be used in teeth where there are aesthetic concerns [209].

The true success of RET is being difficult to evaluate. Regardless of the presence or absence of an intracanal BC, the concentration of irrigating solution, or type of intracanal medication used, different treatment protocols were able to eliminate clinical symptoms and signs of apical periodontitis. Its potential to promote thickening of the canal walls and/or continued root development is, unfortunately, not yet predictable [224].

### 3.2. Regenerative Periodontics

Considered a distinctive tissue structure, periodontal tissue consists of a three-dimensional complex of alveolar bone, PDL, and cementum. The incidence of periodontal disease, the main cause of tooth loss, is increasing among the population, affecting about 20–50% of the global population without being influenced by age or sex [258,259,260,261].

It has a microbial cause and, in most cases, results in irreversible destructive phenomena. Chronic inflammation severely affects the periodontium, leading to the resorption of the alveolar bone, a pathological phenomenon that cannot be stopped by natural processes [257,262].

Nonsurgical periodontal therapies, such as scaling and root planning, represent the first choice methods in preventing disease progression in its first stages, but the removal of pathogens and necrotic tissues provide only partial, local regeneration of the periodontal tissue. Surgery, needed in the advanced stages, or other currently common periodontal therapies, such as growth factors [263] and grafts, could be replaced by the use of SCs as a successful method for treating periodontal diseases due to the existence of SCs in the PDL [264,265].

Since 2004, when PDLSCs were first identified and considered for periodontal tissue regeneration, many other types of stem cells have demonstrated their capacity to form periodontal tissues under certain conditions (Table 2). SC usage has become increasingly relevant in the last decade in the search for an effective solution for periodontitis treatment, despite the fact that regeneration of periodontal tissues is one of the most complex processes in the human body [266]. Thus, aiming high, the target of regenerative dentistry is to develop effective therapies and techniques to treat periodontal diseases using applied tissue engineering and regeneration on the lost or affected support tissue of the periodontium: alveolar bone, periodontal ligament, and cementum [266].

Two major strategies for periodontal regeneration have been outlined: guided tissue regeneration (GTR) and tissue engineering [267]. GTR, a regenerative surgical technique, has been extensively used for periodontium regeneration in recent decades. It aims to prevent apical migration of the epithelium in the bone defect by placing a membrane at the root surface [268,269].

Two types of barrier membranes are used in GTR: non-absorbable and absorbable membranes. The use of the non-absorbable membranes, such as cellulose acetate filters (Millipore filters), rubber dam, specifically processed expanded polytetrafluoroethylene and dense polytetrafluoroethylene has a high risk of infection because a second surgery is required to remove them [270]. Resorbable membranes—such as allogenic soft tissues, freeze-dried skin, freeze-dried duramater, and reconstituted collagen membranes, have been introduced later on—changing GTR into a single-step procedure [270]. The goal of the membrane is to prevent contact between the gingival tissue and the surface of the root, preventing gum growth in the bone space, thereby selectively guiding cells derived from the periodontal tissue onto the root surface. Thus, the periodontal tissue can be regenerated. In practice, a small piece of tissue-like material is inserted between the gingival tissue and the bone [267].

**Figure 4:**
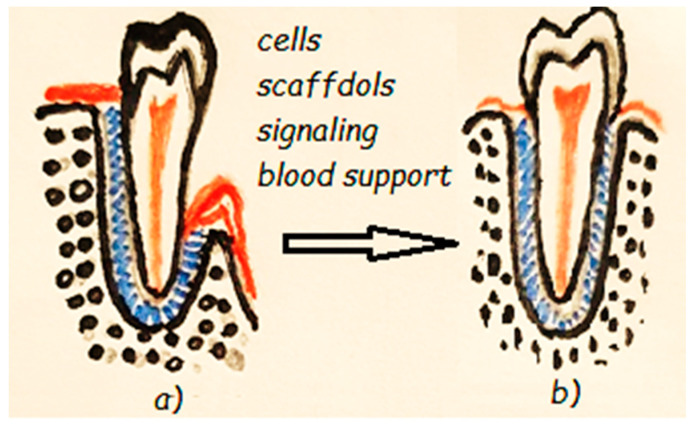
Periodontal tissue regeneration: (**a**) diseased periodontium, (**b**) regenerated periodontium.

Periodontal therapy with SCs has been considered in studies performed on animals, which have reported an effective contribution to the regeneration process of the SCs implanted into periodontal defects [271]. Periodontal tissue regeneration must be viewed as an integrated healing process—a result of the coordinated interaction between stem cells, biomaterials, growth factors, and the particularities of the patient’s immune system (Figure 4). In periodontal regeneration, the tissue engineering strategy may take one of two approaches: scaffold-free or scaffold-based [271]. The scaffold-free approach uses cells or cell aggregates transplanted onto the wound area with no carrier cell. Clinical studies reported that PDLSCs and DPSCs have the potential to form periodontal tissues, but problems occur with cell diffusion out of the defect zone. It has been proven to be a non-relevant regeneration strategy because of the low cell survival rate after transplantation [272]. The cell sheet technique has been developed as a scaffold-free strategy for cell delivery and has been tried in various tissue regenerations, including for periodontal tissue. Cell sheet engineering aims to prevent ECM degradation by isolating cells using enzymes and completely retaining them to ensure normal cell function. Cell sheet engineering can prevent cell migration, but only simple-structured tissues can be regenerated [272]. In conclusion, scaffold-free techniques are not suitable for the complex structure of the periodontium.

For the periodontal ligament, cementum, and alveolar bone complex structure regeneration, the scaffold-based approach is more suitable [273].

Multiphasic scaffolds, with distinctive particularities of each layer both in architecture and chemical/biochemical composition, are required to imitate the complex structure of the periodontium. Additionally, the ECM contributes a 3D substructure for cell adhesion and movement, and contains growth factors facilitating the signal delivery needed for morphogenesis and differentiation. PDLSCs sheets demonstrated the potential of periodontal tissue regeneration in experimental deficiencies in rats, dogs, and pigs [274].

Raju et al. reported successful three-dimensional tissue regeneration of a large-scale tissue injury using bioengineered tissue to simulate the anatomical structure in which two types of cells were used for cell sheet fabrication: rat PDL cells extracted from molars and osteoblast-like cells [275]. Periodontal regeneration with autologous periodontal-ligament-derived cell sheets combined with β-tricalcium phosphate bone was reported as safe and efficacious in a study by Iwata et al. [276].

Biomaterials and controlled drug delivery for periodontal regeneration involve the use of inorganic, polymeric, or composite biomaterials. For bone and cementum repair/regeneration, inorganic biomaterials are the material of choice due to their similarities in composition and mechanical properties. For PDL regeneration, polymeric biomaterials are appropriate. By combining inorganic and polymeric biomaterials, biomimetic scaffolds for bone and cementum regeneration can be fabricated [277].

Thus, bioinspired innovative materials are needed to mimic the complex structure of periodontal tissues at the micro- and nanolevel because, at present, functional periodontal tissue regeneration has yet not been achieved. Many studies with the objective of regenerating the periodontal tissues highlighted that the actual biomaterials cannot exactly mimic the natural architecture of periodontal tissues, so the connections between their components, cementum–PDL–alveolar bone, remain unstable and cannot support teeth or bear occlusal force [278].

For periodontal tissue regeneration, to ensure an ECM-like microenvironment, biomimetic nanofibrous and multilayer scaffolds have been developed. In a recent review, Liang et al. focused on the relevance of advanced bioinspired scaffolding biomaterials and the temporospatial control of multidrug delivery in the regeneration of the cementum–periodontal ligament–alveolar bone complex [267]. A systematic review by Liu et al. [279] presents the newest regeneration developments in the case of all three types of periodontal tissues and for simultaneous regeneration the entire periodontal complex using stem cells, 3D-printing, gene therapy, and layered biostructures.

### 3.3. Regenerative Oral and Maxillofacial Surgery

Oral and maxillofacial surgeries play important roles in the treatment of traumatic and degenerative disease with tissue loss. The techniques used have been improved over time, from using growth factors and platelet concentrates to biomaterial scaffolds and autologous tissues and, currently, SCs in regenerative dentistry. In oral and maxillofacial surgery, tissue engineering and regeneration are the approaches currently available for achieving the goals of reconstruction procedures [280].

In maxillofacial reconstruction, surgeons have two main objectives: to provide the anatomic form and the function of the oromaxillofacial area. Because the facial skeleton has a complex structure, reconstruction should restore the volume, shape, bone continuity, and symmetry of the skeletal bone. Because the numerous soft and hard tissues that form this area provide important functions such as articulation, facial expressions, mastication, swallowing, and breathing, the reconstruction must restore, maintain, and stabilize these tissue functions. In addition, the reconstruction must be performed not only for reconstructive goals but also for aesthetic goals. Hence, different types of tissues must be reconstructed layer by layer [281].

Oral and maxillofacial surgery can use MSCs from the oral cavity, which are an important and easily accessible source to the surgeon. Several maxillofacial bone defects can be approached using bone tissue regeneration. Soft tissue, such as skin and oral mucosa, can also be regenerated [282]. Cartilage regeneration, salivary gland regeneration, fat, muscle, blood vessels, and nerve regeneration represent other applications of tissue engineering in oral and maxillofacial surgery [2,278]. Recent studies highlight the possibility of using GMSCs as the cellular components for 3D bioprinting of scaffold-free nerve constructs needed for peripheral nerve repair and regeneration [283] or for treating gingival defects [284].

#### 3.3.1. Bone Regeneration

Substantial bone defects of the maxilla and mandible, in need of surgery, originate from congenital abnormalities, accidental traumatic injuries, tooth extraction, surgical resection of benign or malign tumors, and infections. The most challenging situation for the maxillofacial surgeon is the restoration of large bony defects due to trauma or post-resection.

In the standard reconstruction of maxillofacial bone defects, autologous grafting is still the gold standard technique, even if it presents many disadvantages [285].

A perfect technique and material for bone reconstruction has not yet been found, even if many clinical approaches have been attempted in recent years. Bone tissue engineering techniques provide a solution for reconstructing large size bone defects in the oral and maxillofacial region using autologous bone grafts, conditioned by adequate vascularization. Wu et al. [286] reviewed new strategies for improving vascularization of engineered bone tissue and their possible feasible clinical applications using SCs, mainly MSCs originating from bone marrow or adipose tissue as well from dental tissues. MSCs are a key element in bone regeneration due to their capacity to induce bone regeneration by mimicking biological processes [287].

After being seeded into newly regenerated tissue, MSCs can be directed to differentiate into osteoblasts which finally initiate the process of mineralization. MSCs can indirectly improve bone regeneration through the secretion of cytokines and growth factors. Two strategies are used: the MSCs are directly transplanted into the defect bone site and combined with an external scaffold; MSCs isolated from the patient and expanded ex vivo are seeded onto suitable internal 3D scaffolds which, in controlled culture conditions, proliferate and pre-differentiate [288]. The most promising is the combination of cells with scaffolds fabricated from different materials and technologies, recently summarized by Chocholata et al. [289]. Several investigations in bone tissue engineering have reported various types of MSCs combined with different scaffolds as potentially suitable for regeneration for surgical procedures in the oral and maxillofacial region [290,291,292,293]. A clinical research study reported biocomplexes fabricated from DPSCs and collagen sponges in human mandible repair with remarkable results [294].

Recently, the use of human GMSCs was considered as a strategy for accidental or trauma surgery treatment, especially for cranial bones. Three-dimensional-engineered scaffolds complexed with GMSCs could provide a new therapeutic approach to improving bone tissue regeneration [295].

Common bone defects in the maxilla and mandible after tooth loss include atrophy of hard and soft alveolar tissue, which result in reduced horizontal and vertical dimensions [296]. In some cases, bone regeneration is required in the atrophic mandible and for maxillary sinus augmentation and dorsal augmentation in rhinoplasty [282]. The atrophic mandible, characterized by a vertical height of less than 20 mm, presents hypovascularity that can determine tooth loss and alveolar processes. The atrophic resorption patterns create important anatomical changes with the risk of soft tissue breakdown and dehiscence as secondary effect of deficiency in blood supply in that area because of the lack of muscle attachments. In Gjerde et al. [297], regeneration of severe mandibular ridge resorption was performed using bone-marrow-derived MSCs, which is a less invasive approach than classical bone grafting. Aspirated from the posterior iliac crest, the bone marrow cells and the plastic adherent cells were expanded in culture medium with human platelet lysate. Afterwards, the cells were inserted into the defect together with biphasic calcium phosphate granules. A significant new regenerated bone formation was induced with a volume appropriate for dental implant installation [297]. Di Stefano et al. [298] tested the effectiveness of enzymatically deantigenated equine bone block as a scaffold during horizontal augmentation of the lower jaw for guided bone regeneration. In addition, they reported the augmentation of a partially edentulous atrophic mandible using an equine-derived block with an expanded polytetrafluoroethylene membrane. The new regenerated bone allowed for a definitive prosthesis [298].

#### 3.3.2. Cartilage Regeneration

The temporomandibular joint (TMJ) is affected by many diseases and defects that can compromise the cartilaginous layer of the condyle. Cartilage is an avascular tissue that has a limited capacity to heal and repair because of limited supplies of nutrients and does not have the availability of blood-borne or perivascular progenitor cells. Many surgical procedures are available for TMJ disorders, but all are aggressive and dangerous for the patient. From simple arthrocentesis to joint replacement, they cannot produce integral regeneration [299].

A recent research objective is the insertion a cell source to manufacture neocartilage after displacement of the dysfunctional disc. Biocompatible scaffolds seeded with cells and biological modulators can be useful in such a process. Thus, the regeneration process of the TMJ is based on several main factors, such as scaffold design and material, stem cells, bioactive agents, biochemical compatibility between the scaffold and the surrounding environment, and the ability of the host to accept the scaffold and facilitate tissue formation [300]. Both natural and synthetic polymers were used for the regeneration of soft cartilage tissue. Collagen, gelatin, hyaluronic acid, fibrin, silk, agarose, polylactic acid, or poly vinyl alcohol are only some of the materials that can be used in cartilage tissue engineering, as reviewed by Jazayeri et al. [300]. Extracting SCs from the synovial capsule surrounding the joint holds has been proven to be a promising choice for generating neocartilage. Recently, Shetty et al. concluded that human DPSCs in porous chitosan scaffolds are useful for regenerating chondrogenic cells [301].

### 3.4. Tooth Regeneration

**Figure 5:**
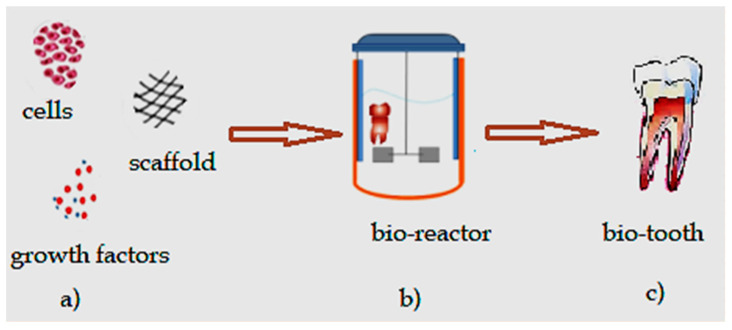
Bio-engineered tooth: (**a**) selection of dental cells sources and scaffolds, with addition of growth factors, (**b**) bio-reactor for in vitro development of bio-engineered tooth bud, (**c**) bio-tooth.

Nowadays, the regeneration of the entire tooth and its replacement represent the final objective of tooth tissue engineering (Figure 5). Even if dental tissues have no capacity for self-regeneration, the teeth are an important source of SCs, offering possible regeneration based on a patient’s own SCs. This technique could be used to create replacements for dental implants and eliminate the risk of rejection, as the new tooth would not be a foreign tissue [302].

Tooth regeneration research using adult SCs has been considered. Autologous DPSCs or postnatal tooth germ cells have limited window of availability, so they can only provide a casual source for whole-tooth regeneration.

The classical tissue recombination has been improved by using collagen drops on the organic culture or by seeding the re-aggregated germ cells on biodegradable polymers. The experiments on animals have shown that tooth-like organs, with dentin and enamel, can be developed by ectopic subcutaneous grafting these cell aggregates under the renal capsule or into the anterior eye chamber [303].

After implantation into the animal’s jaw, a whole tooth could be generated. Ikeda et al. [304] reported successful tooth replacement in an adult mouse, representing an important first step for the transplantation of the bioengineered tooth germ into the alveolar bone to replace a lost tooth. Teeth represent a particular goal for regenerative medicine; they are difficult to recreate due to their complicated structure and numerous functions such as in articulation, mastication, and facial aesthetics. Thus, even if incontestable advancement can be achieved, tooth regeneration based on SCs still has uncertain applicability. Several research studies have reported similar tooth tissues regenerated using different cell types on biodegradable scaffolds, such as silk protein, chitosan [305].

SCs collected from postnatal tooth buds of animals, self-replicated and differentiated in vitro, have been seeded onto a biodegradable scaffold. Their in vivo maturation was achieved by transplanting the seeded scaffolds either into the renal capsule or the omentum, followed by their reimplantation into an extracted tooth place or the jaw [31]. However, by using non-human cells, the chance of immune rejection exists [306].

Ono et al. performed the autologous transplantation of a bioengineered tooth germ in a postnatal canine model and reported functional tooth restoration. The results of the study represented a relevant advancement in whole-organ replacement therapy as well as a practical model for future attempts [307]. Nevertheless, entire-tooth engineering or regeneration is still complicated, and the literature on the subject highlights several problems, such as how to program the stem and progenitor cells to develop into tooth-specific cell types [308,309].

Based on the proven successful applications of SCs in dental tissue regeneration, researchers realize whole-tooth regeneration could be achieved by applying one of two hybrid strategies. The first involves biological PDL and the tooth crown, obtained using stem cells, combined with a metallic or ceramic implant [310]; the second involves a biologically regenerated tooth root combined with a prosthetic crown [311].

## 4. Concluding Remarks and Future Perspectives

In recent decades, major progress has been achieved in regenerative medicine and especially in tissue engineering, which has been used in many clinical applications, but only the first steps toward these goals have been completed [312]. Tissue engineering based on stem or progenitor cells is a promising approach for restoring the integrity of dental and maxillofacial tissues. Research and clinical applications of dental SCs have proven their utility and advantages, such as the capacity for self-regeneration and multidirectional differentiation, easy accessibility, and, importantly, low autologous transplant rejection. However, for real and stable tissue regeneration in dentistry, many other theoretical and technological approaches must be applied in the future for the induction and genetic modification of orofacial SCs. Regeneration of the entire tooth is a major objective for replacing classical dental implants and overcoming their disadvantages. Such an approach would allow the reconstruction or regeneration of teeth in the near future, significantly increasing the quality of dental health.

Future studies are still necessary to identify suitable SCs for performing the physiological role of native tissue, growth factors able to support both cellular differentiation and replication, and to determine the role of microvascularization in tissue regeneration.

## Abbreviations

ECM	Extracellular matrix
SCs	Stem cells
ESCs	Embryonic stem cells
ASCs	Adult stem cells
iPSCs	Induced pluripotent stem cells
MSCs	Mesenchymal stem cells
DPSCs	Dental pulp stem cells
PDLSCs	Periodontal ligament stem cells
PDL	Periodontal ligament
SCAPs	Stem cells from apical papilla
DFCs	Dental follicle stem cells
TGPCs	Tooth germ progenitor cells
SHEDs	Stem cells of human exfoliated deciduous teeth
ABMSCs	Alveolar bone-derived mesenchymal stem cells
SGDSCs	Salivary gland-derived stem cells
OMSCs	Oral mucosa-derived mesenchymal stem cells
OESCs	Oral epithelial stem cells
GMSCs	Gingiva-derived mesenchymal stem cells
PSCs	Periosteum-derived stem cells
PLA	Poly(lactic acid)
PGA	Poly(glycolic acid)
PLGA	Poly(lactic-co-glycolic acid)
PCL	Poly(caprolactone)
HA	Hydroxyapatite
PVDF-TrFE	Poly(vinylidene fluoride-trifluoroethylene)
nBGC	Nanobioactive glass ceramic
RET	Regenerative endodontic therapy
MTA	Mineral trioxide aggregate
AAE	American Association of Endodontists’
EDTA	Ethylenediaminetetraacetic acid
CHX	Chlorhexidine
TAP	Triple antibiotic paste
PRP	Platelet-rich plasma
PRF	Platelet-rich fibrin
BC	Blood clot
GTR	Guided tissue regeneration
TMJ	Temporomandibular joint
